# Improving financial access to health care in the Kisantu district in the Democratic Republic of Congo: acting upon complexity

**DOI:** 10.3402/gha.v8.25480

**Published:** 2015-01-05

**Authors:** Stéphanie Stasse, Dany Vita, Jacques Kimfuta, Valèria Campos da Silveira, Paul Bossyns, Bart Criel

**Affiliations:** 1 Belgian Aid Agency, Kisantu, DR Congo; 2 Hospital of Kisantu, Kisantu, DR Congo; 3 Provincial Health Team of Bas Congo, Matadi, DR Congo; 4 Institute of Tropical Medicine, Antwerp, Belgium; 5 Belgian Aid Agency, Brussels, Belgium

**Keywords:** access, payment modalities, health district systems, regulation, action research, systems thinking

## Abstract

**Background:**

Comzmercialization of health care has contributed to widen inequities between the rich and the poor, especially in settings with suboptimal regulatory frameworks of the health sector. Poorly regulated fee-for-service payment systems generate inequity and initiate a vicious circle in which access to quality health care gradually deteriorates. Although the abolition of user fees is high on the international health policy agenda, the sudden removal of user fees may have disrupting effects on the health system and may not be affordable or sustainable in resource-constrained countries, such as the Democratic Republic of Congo.

**Methods and Results:**

Between 2008 and 2011, the Belgian development aid agency (BTC) launched a set of reforms in the Kisantu district, in the province of Bas Congo, through an action-research process deemed appropriate for the implementation of change within open complex systems such as the Kisantu local health system. Moreover, the entire process contributed to strengthen the stewardship capacity of the Kisantu district management team. The reforms mainly comprised the rationalization of resources and the regulation of health services financing. Flat fees per episode of disease were introduced as an alternative to fee-for-service payments by patients. A financial subsidy from BTC allowed to reduce the height of the flat fees. The provision of the subsidy was made conditional upon a range of measures to rationalize the use of resources.

**Conclusions:**

The results in terms of enhancing people access to quality health care were immediate and substantial. The Kisantu experience demonstrates that a systems approach is essential in addressing complex problems. It provides useful lessons for other districts in the country.

The commercialization of health care by charging fees for the delivery of health care services in the provider's financial interest instead of the patient's interest reduces access to services. It has contributed to the widening of the inequity gap between the rich and the poor. This commercialization has reached dramatic proportions in countries that, by choice or because of a lack of capacity, fail to regulate the health sector and to protect the consumer (1).

Poorly regulated fee-for-service payment systems, being both a cause and a consequence of the commercialization of health services, encourage over-servicing for those who can afford to pay or whose costs are met by pooled funds and under-servicing for those who cannot pay (2). Indeed, fee-for-service payment encourages inefficient provider behavior and generates inequity (3). It allows considerable lack of transparency, makes health costs unpredictable for patients, and sometimes causes catastrophic expenses. Therefore, the commercialization of health care and fee-for-service payment is intrinsically linked, and it initiates a vicious circle in which access to quality health care gradually deteriorates.

International literature is rich in reflections and experiences that aim to improve access to quality health care and to protect the patient from fee-for-service payment abuses and from the commercialization of health care (4–6). Abolishing user fees is high on the international health policy agenda. However, the sudden removal of user fees may also have disrupting effects on the health system, such as dramatic reduction of financial flows at the facility level, reduction of the quality of services, disruption of the referral system, and demotivation of the health staff (7–13). Moreover, in resource-constrained countries, such as the Democratic Republic of Congo (DRC), exempting the poor from payment of fees would mean that virtually everybody must be exempted from payment. Today, such a policy is neither affordable nor sustainable in the DRC, even with substantial donor aid.

In its health program in the DRC, the Belgian bilateral development aid agency (known as the Belgian Technical Cooperation, BTC) aims to contribute to the implementation of the National Health Development Plan (PNDS, *Plan National de Développement Sanitaire*) (14) through a combination of financial and technical support. This plan is expected to improve access to quality health care in pilot health districts. Prominent in the BTC approach is the intention to foster a process of systematic learning from these pilot experiences to feed national policies in a bottom-up fashion.

In one of these pilot districts, the district of Kisantu, the BTC concentrated on reducing user fees by applying a subsidy at the general hospital (GH). However, a number of conditions were attached to such a subsidy: 1) the change from a system of fees for each individual service to a flat fee and 2) the requirement that the patient be referred by a peripheral, public-oriented health center within the district boundaries (except for a number of listed emergencies). With these conditions, the new system was expected to contribute to rationalize the functioning of the local health system, improve access to quality health care, and enhance appropriate utilization of care.

Although other districts supported by the BTC in other provinces of the DRC also implemented similar changes into the health system, there were substantial differences such as the application or not of a subsidy, the type of services included in the flat fees, the monitoring modalities of the interventions, the geographical and cultural context. Initially, such a study was not planned within the BTC health program. It was developed under the initiative of the local actors, the backstopping team, and the technical assistant, and it did not require a specific budget. This is why the study was undertaken only in the district of Kisantu.

## Objectives

This paper aims to present a detailed account of the BTC intervention in Kisantu. The intervention implemented deep reforms in the local health care delivery system with the intention of improving people's access to care at the appropriate level in the local health system. More specifically, the following objectives are set forth. First, we will provide a comprehensive description of a reform induced by the intervention of BTC in terms of access to health services and the various changes incurred in factors related to it. Second, we will describe in detail the process of change in Kisantu during 2008–2011 (i.e. describe *how* change was introduced). We will describe the managerial processes used in the planning, implementation, and evaluation of the various changes. Special attention is given to the process of team building of the district executive, which involved health workers at different levels of the local system. Third, we will discuss the relevance of this particular ‘case’ for the national Congolese health system. What have been the conditions of success of this reform experience? Why and how can this local experience be useful for the country as a whole? Finally, we will discuss whether the shift from fee-for-service payments to flat rate payments, paired with a wide range of accompanying reorganization and rationalization measures, might be a realistic policy option for countries that face financial problems similar to those of the DRC; that is, in their endeavor to evolve from quasi-generalized and poorly regulated fee-for-service payments to financing systems with more solidarity between patients, and with a higher degree of pooling of financial contributions.

## Context

The DRC has the legacy of a well-organized and functioning district primary health care and referral system. However, the situation in the health sector, and in all other social sectors, has dramatically deteriorated in the past two decades. Although the Ministry of Health in Kinshasa has not been very effective in recent times in trying to turn the tide, it has managed to clarify the structural organization, functions, and norms of the district health system.

The DRC is divided into 11 provinces. Each province is divided into health districts, which in turn are divided into health zones (*zones de santé* in French). A health zone in the DRC corresponds to what is internationally referred to as a health district (15). For the purposes of clarity, the term ‘district’ will be used in this paper to refer to the Congolese health zone, that is, a network of primary health care facilities, each of which serves a well-defined part of the district area/population, and a GH, which acts at the referral level for the entire health district area. The facilities can be owned by the State, or by not-for-profit private actors, the latter generally having established a contractual agreement with the State clarifying the rights and duties of both partners, but remaining largely autonomous in terms of management. Since colonial times, religious missions have acted as major implementing agencies in the health sector, and the DRC now also has a thriving not-for-profit indigenous network of religious health care providers who are major partners in the management of the district health system. State and private (religious or other) not-for-profit first-level facilities, labeled Integrated Health Centers (IHCs), are in principle under the authority of the local District Central Office (the *Bureau central de la Zone* in French), headed by the District Medical Officer (the *Médecin Chef de Zone* in French). Conversely, Non-Integrated Health Centers (NIHCs) are not subject to any agreement with the State and are usually profit oriented. However, supervision by the district team is often limited to mere data collection and establishing drugs inventories. Moreover, when the IHC or GH belongs to a private actor, supervision is considered intrusive and unnecessary and is usually absent.

In 2008, the DRC was ranked sixth on the list of failed states because of its inability to provide public services, erosion of legitimate authority, corruption, criminality, and involuntary movement of populations (16, 17). In 2011, the United Nations Development Programme ranked the DRC last in human development among a list of 186 countries and territories (18). In the DRC, disengagement of the State from the regulation and financing of the health sector, in addition to governance problems, has resulted in a profound weakening of the country's health system. Unregulated fee-for-service payment is widespread and is both a cause and a consequence of the commercialization phenomenon, which is gradually depriving both urban and rural populations of access to primary health care.

In some areas of the country, the public health system has virtually collapsed and health care delivery is largely left to informal private providers. The public health budget (3% of the annual state budget in 2008) serves mainly to finance irregular and very low salary payments to government health workers (19–21). Unregulated fee-for-service payment by patients is widespread and renders the cost of care completely unpredictable for the patient. Direct payment is requested for every single intervention, be it the administration of an injection or the demand for a laboratory examination; drug prescriptions are quite irrational and access to quality care is poor, especially in district hospitals, which are associated with higher costs than health centers. The disengagement from regulation also concerns universities and schools that train health workers. These training institutions have been booming in the past decade, and health facilities are steadily increasing in number every year. As a matter of fact, in 2008, 470 Technical Medical Institutes and Medical Teaching Institutes (*Instituts des Techniques Médicales*-ITM and *Instituts d'Enseignement Médical*-IEM, respectively, in French) were known, equivalent to an increase of 84% in relation to 1998. Yet, 108 Higher Technical Medical Institutes (*Institut Supérieur de Techniques Médicales*) and 39 Faculties of Medicine (an increase of 103 and 130%, respectively, in relation to 1998) were also known. Only in 2009, 25,916 nurses graduated exceeding the need of the planned health districts. In the same year, over 2,000 doctors graduated from the three ‘traditional’ universities of Kinshasa, Kisangani, and Lubumbashi (22–24). The quality of education that these institutions provide is poorly regulated. For example, in 2009, 56% of the ITMs were functioning without official approval decree (24) Due to brain drain, aging staff and demotivation, there is an evident lack of competent professors contributing to the decrease in quality of education (25). Many universities, institutes, or training schools clearly look for profit and produce as many medical staff as possible. These practices create a plethora of human resources, of doubtful quality throughout the country. The situation is further aggravated by the retirement conditions. Indeed, given the very poor pension allowances provided by the State, retirement signals the end of topping-ups and informal remuneration, without which the health worker cannot survive.

The overall result is poor access to health care and huge unmet needs regarding care in large parts of the country. Reasonably decent health care is being offered in scattered ‘islands’ (i.e. in settings that feature some level of external support from bilateral or non-governmental organizations). The DRC is attempting to rebuild its health system; in 2006, the nation elaborated their Health System Strengthening Strategy (SRSS, *Stratégie de Renforcement du Système Sanitaire*), which aims to reorganize the health system to improve access to quality health care (22, 23). However, a large gap remains between governmental policies and the realities on the ground.

## The District of Kisantu

**Fig. 1:**
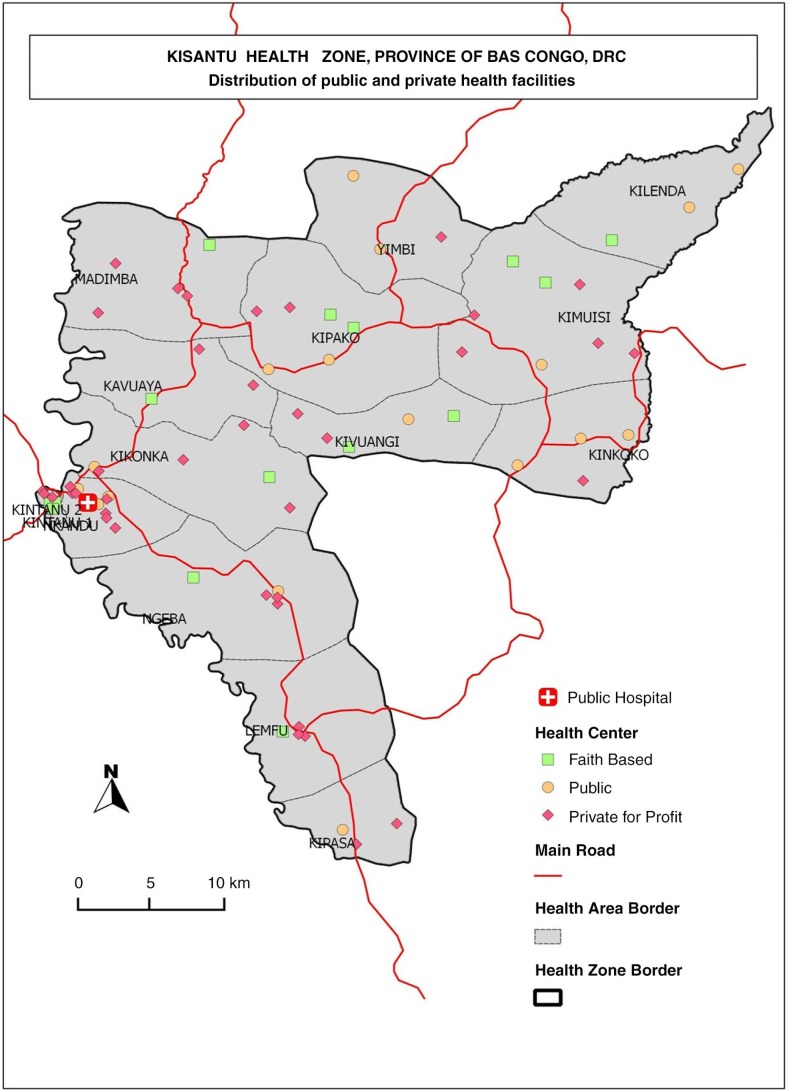
A map of Kisantu district (health zone), Bas Congo, DRC.

Kisantu district is located in the province of Bas Congo in the extreme southwest of the DRC and counts 144,395 inhabitants, among which approximately 45% live in the town of Kisantu and its surroundings (Fig. 1). It has one GH located in the semi-urban zone, as well as 16 IHCs. The GH belongs to the Diocese of Kisantu; however, it has signed an agreement with the State and operates as a district-designated hospital, assisting the government in providing healthcare for its population. Four of its seven resident medical doctors are also priests, and each year the hospital welcomes approximately eight freshly graduated doctors who will train at the GH during a stay of 6–24 months. Four IHCs are located in the vicinity of the GH (i.e. less than five km distant). Kisantu has a long history of cooperation with Belgium. In the 1970s and 1980s, it cooperated with other health zones to feed and influence health policies throughout the DRC (26, 27).

In 2007, a small local health insurance scheme was set up (*Mutuelle de Santé de Kisantu* – MUSAKIS); it consisted of approximately 1,000 members in 2008.

As in most other Congolese districts, the Kisantu General Hospital was not really playing its referral role. It was actually competing for patients with the first level of care in the local health system. ‘Complacency’ outpatient consultations at the hospital by the better-off were frequent, and represented an important income opportunity that was difficult to obtain in the inpatient departments. Therefore, hospital staff would prefer to turn away from inpatients in order to look after outpatients – whether they were referred or not – and compete with the health workers operating in gradually deserted IHCs.

In 2008, the 270-bed hospital with over 200 employees (52% medical staff, 48% administrative or maintenance staff) had a bed occupancy rate of only 53.3%. This figure illustrates the general glut of staff in Congolese health facilities. The hospital admission rate for the population living in the district was 22‰; the utilization rate at first-level facilities (IHCs and GHs combined) was 0.4 new cases/inhabitant/year; 85% of the expected births in the district were attended by qualified staff in an IHC or the GH; among those births, the district maternal mortality ratio was 192/100,000. The patients referred to the GH by the IHC network constituted only 7% of all outpatient department consultations at the hospital; only one-fifth of consultations were eventually hospitalized. The reasons for these low utilization rates and for this high maternal mortality ratio are complex and multiple. The lack of geographical accessibility due to road conditions is a major component of the problem, as are the local health services’ lack of psychological and cultural accessibility. However, in our view, the primary constraint on access to health care is financial in nature.

In 2008, less than one-half of all patients admitted to the hospital (42.8%) were able to pay their bills. This situation clearly had an important impact on the hospital's ability to pay its staff, as topping up (pay supplementation which was added to the State salary and which was paid with the GH income) represented 75% of total staff remuneration. Thus, health workers facing insufficient resources were increasingly tempted to develop informal payment systems in a context of poor regulation and control of such practices. Short hospital stays are expected in a hospital that competes with first-level care facilities. However, the average length of stay at Kisantu GH was quite long (9.4 days), likely because patients’ difficulties raising money at every single step of their itinerary in the institution resulted in extended stays.

## Methods

In terms of ‘positionality’ (28), the first author (SS) of this paper is an insider in the local health system. She worked as a BTC technical assistant in the DRC from 2008 to 2011, and was based in Kisantu. In that position, she had many opportunities to interact with policy makers at the provincial and central levels of the system. These interactions were facilitated by the relative proximity of Kisantu to the capitals of the Bas Congo Province (Matadi) and of the country (Kinshasa). The other authors were colleagues working in the Kisantu district (DV, JK) or were involved in coaching the local reform process (PB, BC), or contributed to the writing process of this manuscript (VCD). The objective of the BTC's intervention in Kisantu was to improve access to quality health care through a set of processes that could eventually be used elsewhere in DRC. The details of the intervention were developed progressively on the basis of a comprehensive situation analysis (29) and were implemented in close collaboration with a range of local and external actors to the district (Congolese and expatriate public health specialists). Funding from the BTC also enabled input from external consultants on an *ad hoc* basis.

**Fig. 2:**
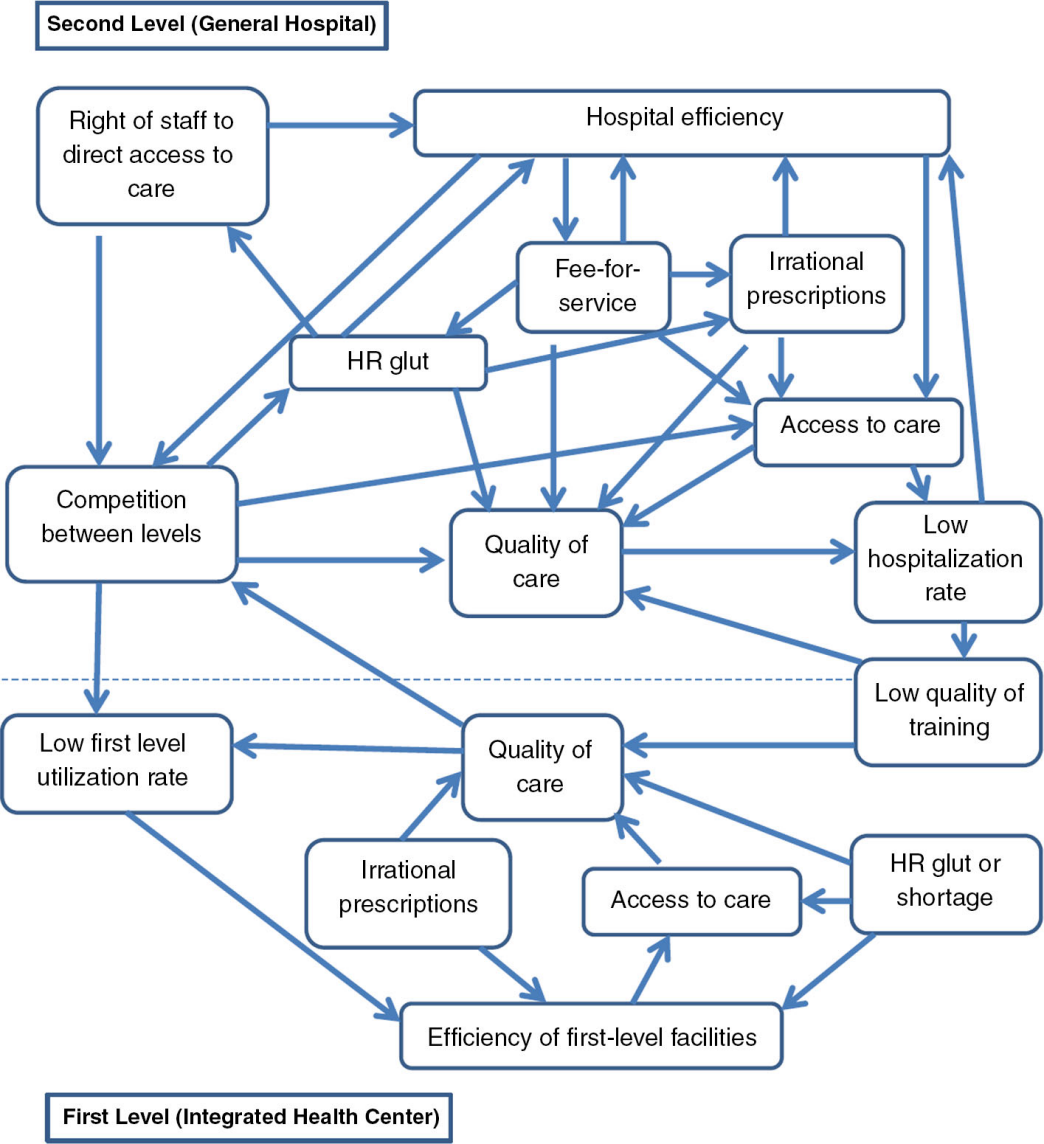
The health district as a complex system.

The method used was action research, an iterative process of problem analysis, hypotheses generation, identification of solutions, implementation, and evaluation of the action(s) taken (30–33). Action research is appropriate for studying complex problems (such as health care access) in complex systems (such as the Kisantu local health system) as it takes into account the various interrelations with various actors in the system, the context specificities, the connections between actors, context and the system itself, and unexpected results. Lessons learned during the action-research implementation, models, and frameworks used are source of inspiration to other wanting to develop or scale-up similar experiences. From the start, the Kisantu intervention considered the health system as an open complex system with interlinked components interacting with the context in which the health system is located. Understanding this interconnectedness and complexity is at the core of a systems thinking approach that views the system as a whole, with properties beyond the component parts (34). In this perspective, which is represented in Fig. 2, the application of an action to one component of the system can upset the balance of the whole system.

However, the analysis of such complex systems is a dynamic and continuous process, and all components cannot be identified or understood at once. Decision-making, action implementation, and their sometimes unexpected and unintended consequences gradually led to a deeper understanding of the system, and new elements and dimensions were progressively integrated into the reform process in order to maintain the system.

The description and analysis of the Kisantu reform process is based on a number of sources. First, there is the vast amount of routine data collected through the district's health information system (e.g. monthly data about total inpatients, outpatients, first-level curative care utilization rate, GH bed occupancy rate, …). The data refer to the financing of the system, as well as to the volume, nature, and flow of health services utilization. Second, there are additional data that were not routinely available, but were specifically collected by the project team at the level of the IHCs and the HD to enable the comprehensive and continuous monitoring of the intervention and its results. For instance, additional data such as the origin of patients (coming from the district of Kisantu, another district of in the province of Bas Congo, or from outside the province), the reference from a health center within or outside the district, analytical accountancy with identification of the patient's and the BTC contributions to the general monthly income of the GH. Those additional data allowed, for example, to understand whether the increase of the GH bed occupancy rate was caused by an increase of the number of patients coming from the district of Kisantu or from outsiders. Finally, the personal field notes of the Kisantu-based BTC technical assistant and various reports from external consultants during the period of 2008–2011 constitute a third source of data.

Data were easily available as the same investigators developed and controlled research tools, trained staff to use them, checked for their availability and reliability.

The Kisantu reform process has doubtlessly benefited from a level of financial and other resources far beyond what is routinely available in other Congolese districts. Such input was deemed justified because of the ability to systematically relay the lessons learned in this pilot setting to the provincial and national levels and to share the experience with other district executive teams. The total amount of financial support by the BTC to Kisantu during the 4-year period (from April 2008 to April 2012) was 2.35 million Euros. The BTC has invested approximately 0.85 million Euros in infrastructure and equipment, about 1.1 million Euros in the provision of subsidies for hospitalized patients and for the retirement of staff, about 50,000 Euros in training, and some 350,000 Euros for external consultancies. Given its encouraging results, Kisantu GH benefited, in 2013, of another million Euros for building and equipping new surgical and intensive care units, new x-ray and laboratory departments. Retirement of personnel in the IHCs and the GH, based on age of the person or number of years of service and according to the national code of labor in DRC, was updated every year by the district. It is important to highlight that the human resources rationalization process as well as the maintenance of infrastructure and equipment need continuous investment without which the acquired results cannot be sustainable.

## Results

In this section, we present a detailed and chronological description of the process of change that took place in Kisantu, evolving from an initial in-depth situation analysis to action planning, implementation, and evaluation. The action planning was consistent with the overall purpose of the intervention: to improve people's access to quality health care at the appropriate level in the local health system. The process followed was by no means a linear one. New elements that surfaced during the course of the process, especially those pertaining to the perceptions and behavior of local stakeholders, were taken into account whenever relevant, leading to a flexible and iterative process of change. The action taken was not limited to one particular element of the local system; it consisted of a variety of concomitant actions implemented at different levels within the system.

**Fig. 3:**
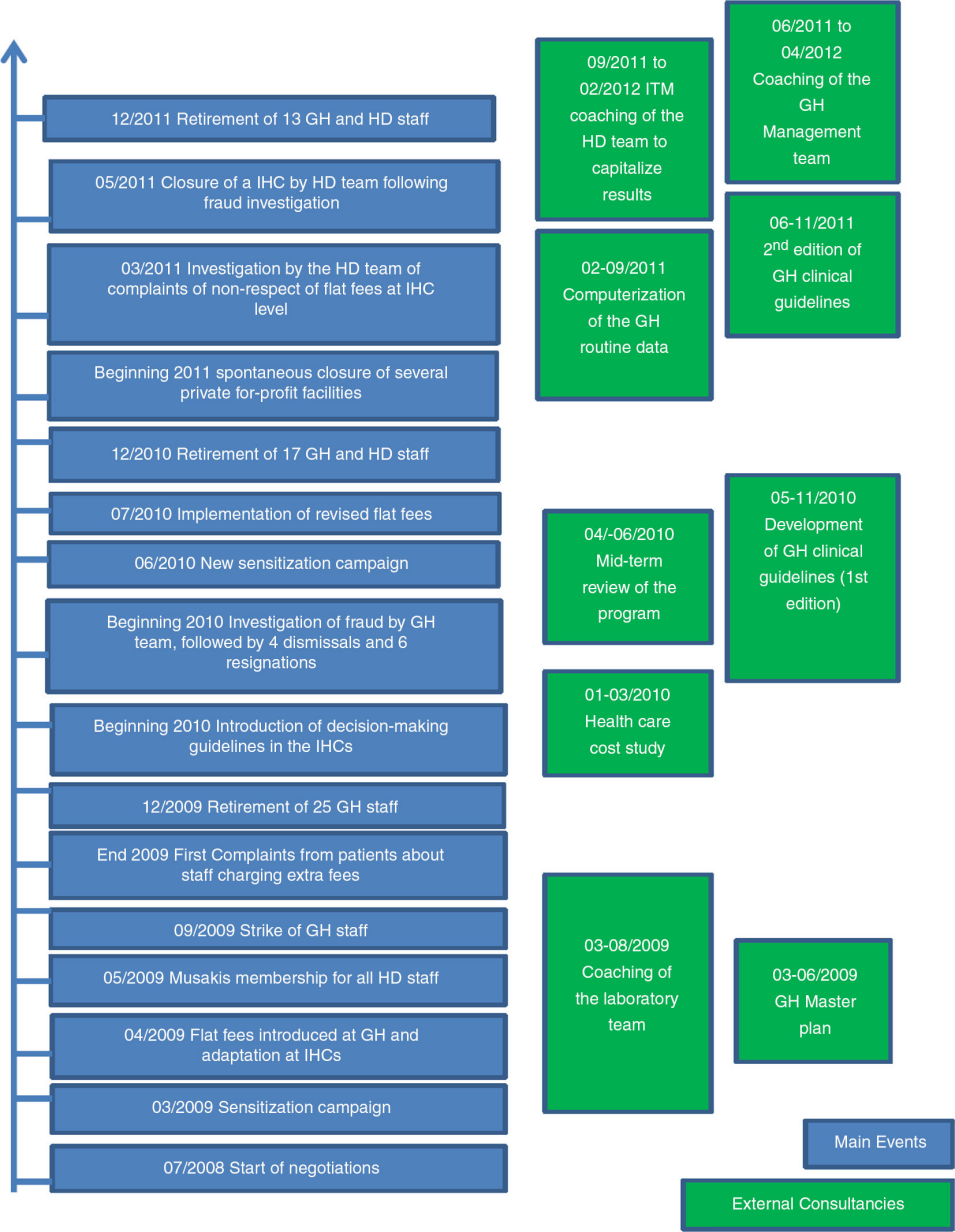
Timeline. Source: Project data, 2008–2011.

One of the first changes introduced in Kisantu, and very much in line with the SRSS, was the launching of a ‘renewed’ District Health Executive Team composed of staff from both hospital and district services. Instead of having distinct management entities for the hospital and the district, a single structure was created. It required quite some time before this team became fully operational and effective in exercising leadership over the local health system. Many discussions and meetings were necessary during the initial months for the newly built team to endorse the critical analysis of the existing situation. The first decisions were taken on a consensus basis after almost 1 year of negotiations. Resistance to change, difficulties for two previously independent entities to work together, and loss of unofficial income and privileges for many of the staff were among the most likely reasons why the process took so long. Figure 3 presents a timeline that describes the main steps of the overall reform process.

In April 2009, the following four decisions were taken by the executive team:

1. Implementation of flat user fees at the hospital replacing the former fee-for-service system. The flat fees covered the consultation (and/or the hospitalization), laboratory examinations, echography, and/or X-ray and medicines for the entire episode of disease. The fees were calculated on the basis of yearly local financial audits organized by the Diocese of Kisantu but also in reference to the National Health Accounts of the DRC (2008);
2. Subsidy of the flat fees charged by hospital for patients who lived within the district boundaries and had been referred to the GH by an IHC (40–75% of the flat fees paid with BTC funds with a maximum subsidy for women and children health care);
3. Subsidized retirement (with BTC funds) of approximately 25 hospital staff by the end of 2009;
4. *MUSAKIS* membership for all hospital staff, with registration premiums paid by BTC funds for the first year.

Financial barriers were much more pronounced at the GH than at the IHCs. The decision to subsidize the GH alone was the result of BTC budget constraints.

Regular meetings of the unified district executive team were scheduled. The initial results of the reforms (positive or negative, expected or unexpected) surfaced little by little, and were promptly discussed at these meetings. The need to follow the reforms, and to adjust action whenever necessary, was at the core of most discussions. Initially, the executive team did not insist strongly on the need to rationalize diagnostic and therapeutic behavior because of the anticipated resistance from hospital-based medical doctors. However, the team was aware that the flat fee structure was decided on an approximate basis, without the necessary detailed financial information (for this reason, a cost study was planned in 2010).

**Fig. 4:**
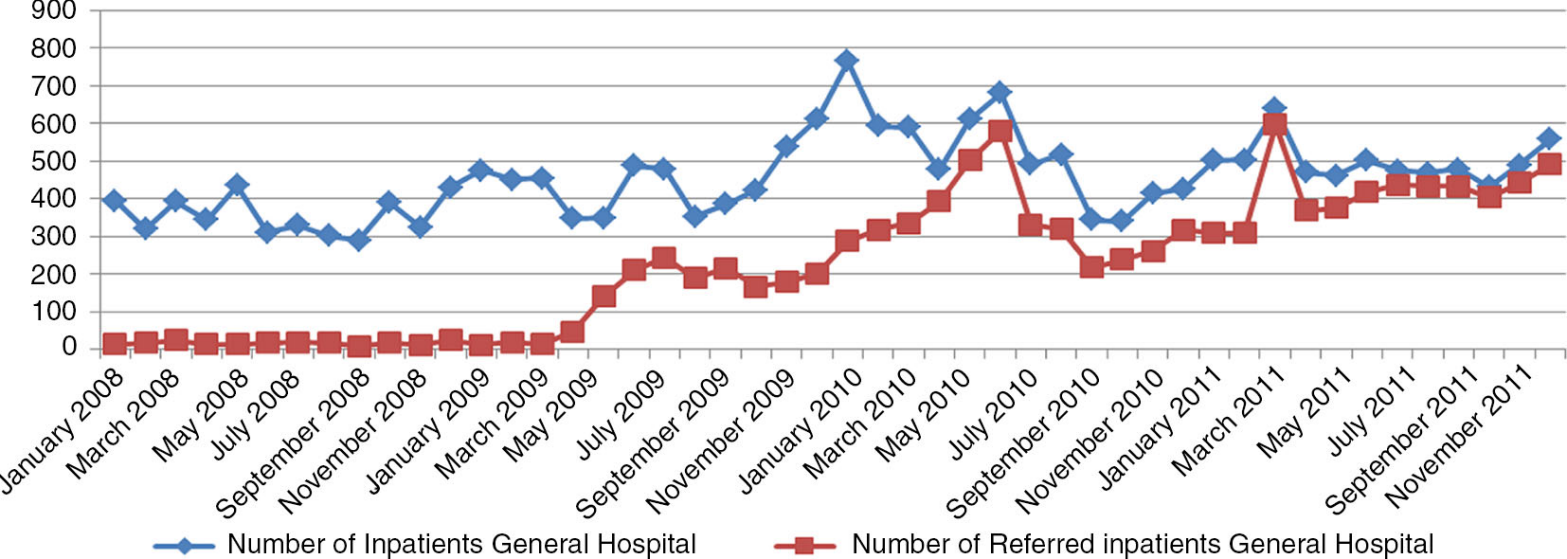
Evolution of the inpatients: Kisantu GH 2008–2011.

**Fig. 5:**
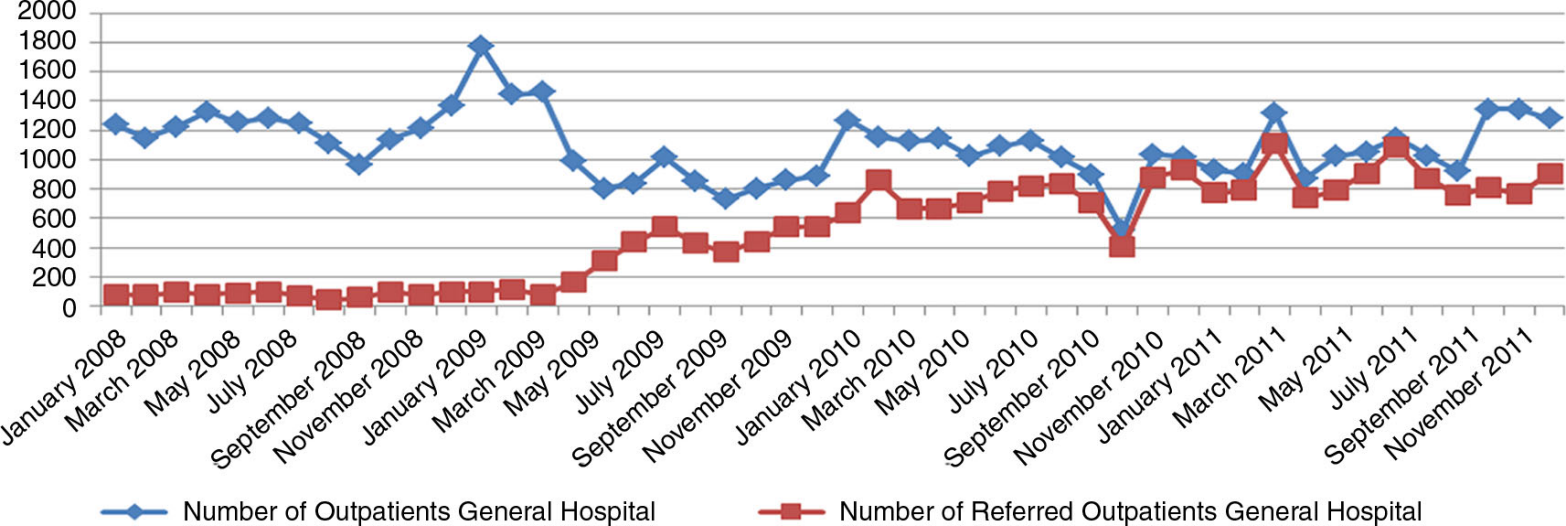
Evolution of the outpatients: Kisantu GH 2008–2011.

**Fig. 6:**
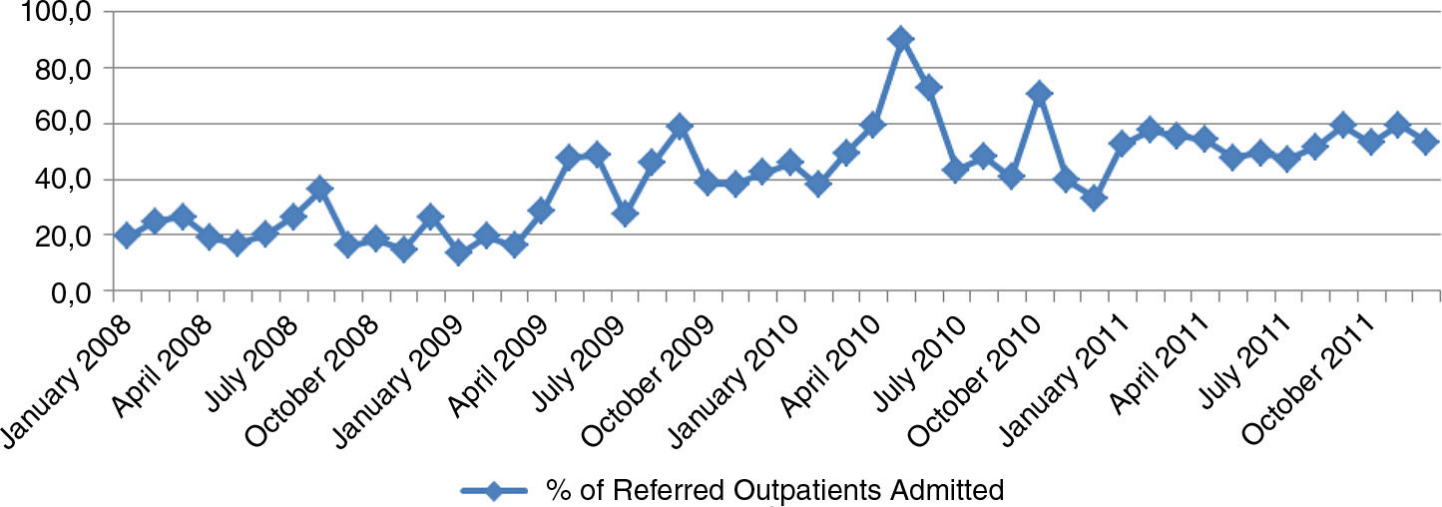
Evolution of admissions among referred outpatients: Kisantu GH 2008–2011.

In March 2009, information and sensitization activities regarding the reforms were conducted through television, radio messages, advertisements, discussions with auditors, and meetings with health staff. In April 2009, the new flat fee structure was applied at the hospital level. Its effect on the utilization of health services was immediate (Figs. 4–6): the use of curative consultations as first-line care increased, whereas the number of outpatients at the hospital level decreased, and the volume of (referred) inpatients increased.

**Fig. 7:**
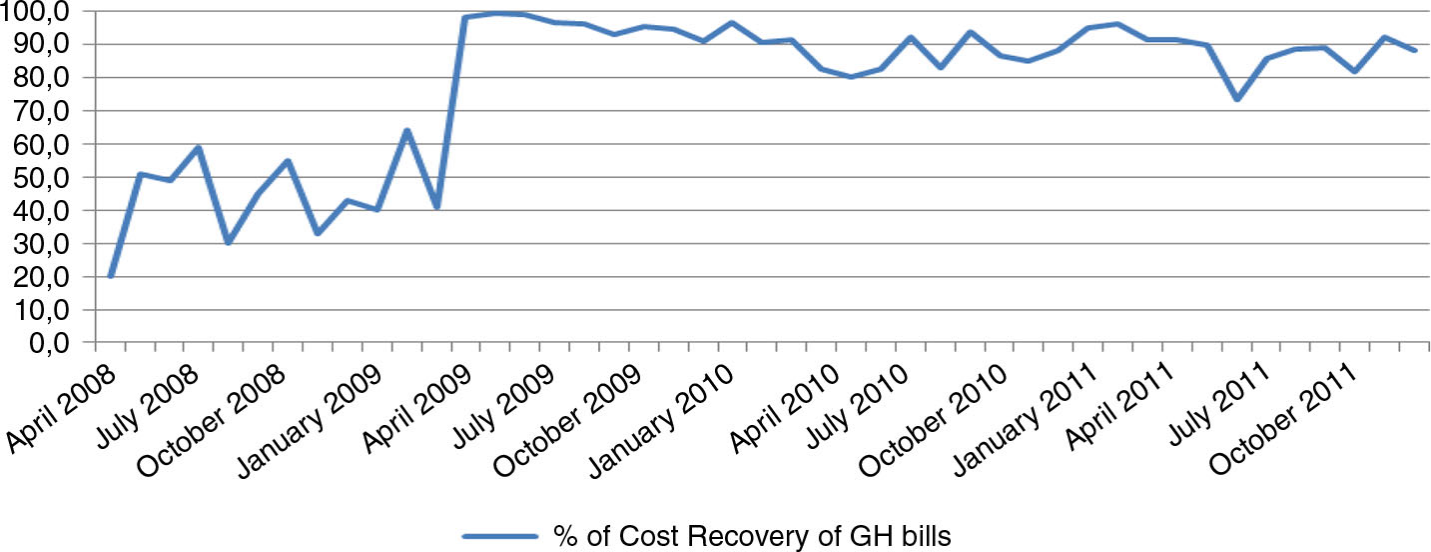
Evolution of the cost recovery rates for hospital bills.

The introduction of decision-making trees at the health center level increased the rationality of prescription behavior. More than 90% of patients presenting at health centers in 2010 were prescribed essential drugs; this proportion had been less than 50% in 2008. No change in prescription behavior was observed at the hospital level in 2009. Although hospital finances gradually improved, a shortfall remained because 10% of patients remained unable to pay their health care bills despite the subsidies (Fig. 7).

Fraud (e.g. health center staff selling referral bills to patients) was observed in some health centers, especially centers located close to the district hospital. To address this problem, the executive team decided to reinforce supervision. By September 2009, an ombudsman was appointed at the hospital to collect patient complaints. Some months passed before victims of fraud (the staff charging extra fees) dared to complain. So as not to discourage potential victims, the executive team took the complaints very seriously, led a number of investigations, and took disciplinary action when necessary. In early 2010, four staff members had been dismissed because of fraud and six other had spontaneously resigned.

In addition to fraud, a lack of understanding of the reforms also led to mistakes. For example, some staff continued to charge patients for dressings and gauze because they were convinced that this was not included in the flat fees. New sensitization and information activities were clearly needed. These activities were organized in the months after the start of the reform process, and targeted the medical staff in particular.

Despite the regular payment of staff salaries and the repeated information campaigns, it rapidly appeared that the previous informal (and illicit) revenues were higher than the new wages paid to staff along the lines of a transparent salary structure. This loss of income was a source of great discontent among hospital health workers, especially among nurses. In September 2009, hospital staff went on strike and petitioned for higher monthly remuneration. In 2010, salaries were increased in accordance with government (theoretical) recommendations, which have rarely been applied elsewhere. The BTC helped the hospital to pay its staff and covered the shortfall caused by insolvent patients. This increased expenditure for staff wages was eventually to be integrated into a revised flat fee schedule.

Several attempts were made to obtain support at the provincial and central levels and to integrate the results of the reforms into national health policies; however, the response has been poor. Although some elements of the Kisantu experience, such as the implementation of flat fees and the use of decision-making trees and hospital protocols, were incorporated into the new version of the SRSS, the first field visits from the Ministry of Health in Kinshasa only took place in 2011, despite repeated invitations. However, on a positive note, we must mention that several executive teams from neighboring districts or from other BTC health projects visited Kisantu to learn from it. The BTC contributed by organizing six exchange visits in 2010 and 2011.

A hospital cost study was organized in 2010 by a local consultant who had been hired by the Diocese. Based on the more accurate knowledge of exact costs, the flat fee structure was upgraded; the BTC subsidy was increased further to keep from increasing the part to be paid by the patient. New sensitization and information activities were organized through radio and television, as well as through community representatives.

A new wave of retirement of health workers was organized by the executive team and financed by the project in 2010 and in 2011. Personnel were redeployed among health centers and the hospital; new personnel were recruited where needed. Overall, the human resources in Kisantu district passed from over 200 staff in 2008 to 140 in 2011 with an increase in the proportion of paramedical and medical staff from 52% in 2008 to 60% in 2011.

In 2010, despite many meetings with the resident medical doctors, their prescription of drugs and ordering of laboratory examinations remained highly problematic. Approximately 30% of the prescribed drugs were still specialties, most of which had to be purchased outside the hospital, and there was ample evidence of prescriptions containing four or even five antibiotics, not corresponding to any evidence-based protocol. To address this problem in a non-threatening way, the services of an external consultant were employed to discuss the rationale of the current prescription behavior and to develop, together with the medical staff of the hospital, a set of evidence-based guidelines that were adapted to the local context and included the (limited) existing national protocols. The process took approximately 6 months, but was met with high interest and participation from the hospital doctors. The effect of the newly introduced guidelines was impressive: in 2011, the resident medical doctors prescribed essential drugs in 90% of cases and all antibiotic therapy was consistent with the guidelines. These guidelines were also shared with the provincial and central health authorities for use in other districts and provinces – a source of pride among the Kisantu hospital staff.

The year 2011 was also marked by the disappearance of several private for-profit health centers because of a lack of patients. Such an event would have been unthinkable in 2008, as the private for-profit sector seemed to be unwavering. It is an indicator of the enhanced accessibility and acceptability of the public and private not-for-profit health care delivery system. In the same year, several smaller health posts with a limited curative care workload were shuttered because they did not respect the reform guidelines. The latter decision was an indicator of the enhanced decision-making power of the district executive team. Table 1 presents a selection of indicators pertaining to the functioning of the district health services system over time.

**Table 1:** Evolution of indicators

	2008	2009	2010	2011
General hospital bed occupancy rate (270 beds)	53.3%	69.3%	74.9%	85.1%
First-level curative care utilization rate (new cases/inhabitant/year)	0.41	0.51	0.56	0.50
Hospitalization rate (inpatients/population×1,000)	21.2‰	27.5‰	34.4‰	32.7‰
Average length of stay (without orthopedics)	8.8 days	8.1 days	8.1 days	7.6 days
Total of new cases at the hospital outpatient department	14,529	12,468	12,424	12,282
Outpatients from the district, %	77.1	78.7	81.3	82.9
Referred outpatients, %	7.8	36.8	79.6	89.8
Hospitalization among referred outpatients, %	21.7	39.9	50.5	53.3
Inpatients coming from the district, %	74.0	78.3	85.4	86.8
Referred inpatients coming from the district, %	6.1	35.0	76.1	93.9
Proportion of recovery of patient bills	42.8%	83.9%	87.2%	87.8%
Autonomous staff incentives at independent health centers (USD)	86,601	90,874	105,581	127,843

Source: Kisantu District Hospital and Kisantu district records and monthly statistics.

The GH bed occupancy rate increased from 52% in 2008 to more than 85% in 2011, and the proportion of referred inpatients increased from 6.1% in 2008 to 93.9% in 2011. To ensure that those changes are indeed related to patients from the Kisantu district and are not caused by an increase of patients coming from cities such as Kinshasa or Matadi (where health care is much more expensive and business-oriented), the hospitalization rate based on the origin of patients (inpatients living in the Kisantu district/Kisantu district population×1,000) was also followed, and the data demonstrate a 54% increase between 2008 and 2011. This trend is confirmed by the increase of the percentage of inpatients coming from the district from 74% in 2008 to 86.8% in 2011. The first-level curative care utilization rate in terms of new cases per inhabitant per year also increased, while the total number of new cases at the hospital outpatient department decreased. This finding shows that patients who previously used the GH services directly changed their behavior and went first to an IHC, where a proportion of them could be treated without any need for referral. One indicator designed to follow the relevance of referral from the IHCs to the GH was the percentage of referred outpatients that became admitted patients, which increased from 21.7% in 2008 to 53.3% in 2011. This result can be explained by the fact that the reforms have led to more transparency in the payment system, better availability of drugs, higher predictability of the costs to the patient, and better financial access to health services at the hospital level through the conditional subsidy. To illustrate this, the proportion of recovery of patient bills totaled 87.8% in 2011, up from 42.8% in 2008. The results also indicate a more rational use of resources, with improved gate-keeping by the first line and more efficient use of the hospital.

## Discussion

Our first two objectives were to provide a comprehensive description of the changes induced by the intervention in terms of access to health services and changes incurred in the various factors related to it; and describe the process of how change occurred. Results presented in the previous section shows that access to health care has improved at both first and second levels of care in the Kisantu district health system.

The decision to subsidize only the GH and not the IHCs was due to budget constraints, as well as to the underlying hypothesis that the expected increase of patients at the IHCs would generate an increase of income at this level and would allow the staff to improve their revenue. The autonomous staff incentives, which represent a topping up of the official salaries (official salaries are approximately 20 USD per month for a nurse), are the available amount in their cashbox after all necessary expenses (e.g. drugs to re-stock the pharmacy, maintenance) have been made. For all IHCs combined, these incentives increased from 86,601 USD in 2008 to 127,843 USD in 2011, which represents an average increase of topping up of 47.6%. Although part of this official increase that replaces previous unofficial income is very difficult to assess, most of the in-charge nurses at the IHCs recognize that their total income had indeed increased. The greatest increase was observed at the IHC that was closest to the hospital.

The management of the reforms was centered on the systematic use of a cyclical process of situation analysis, problem identification, implementation of solutions, and evaluation of the new situation, process characteristics of action research and learning cycles. This process was fed by data from the routine health information system, and enriched with observations made by the executive team members during their daily activities at the GH and IHCs. The executive team progressively increased its understanding of and grip on the complexity of the system, which helped enhance its leadership. The meetings of the executive team were centered on new problems to be addressed and solutions to be implemented. It rapidly became clear that the managerial approach of such complex reforms needed to be dynamic, iterative, and flexible so that the action taken, together with the sensitization campaigns that accompanied it, could be adapted to a changing situation. Hence, there was a need to implement an information monitoring and reporting system that included qualitative information about critical incidents. In turn, such a system implies the close supervision of activities at all levels of the health system and an integrated executive team with room for supervisors to present and discuss their findings.

As related to the relevance of this ‘case’ to the Congolese health system, our third objective, due attention was given by the district executive and BTC teams to regularly inform the provincial and national authorities about the changes that were taking place in Kisantu. The executive team has repeatedly invited the provincial and the central levels to visit Kisantu district, its IHCs, and its GH in order to share the implemented process and obtain feedback from the authorities. The team was also invited to present their experience at the first Regional Workshop on Health Systems Strengthening in September 2011, in Kigali. In addition, as mentioned above, several districts, both inside and outside the Bas Congo Province, have visited Kisantu and employed the lessons learned from its reform process.

However, we have observed that exchanges inside the DRC had only a limited impact in terms of involvement from the central and provincial levels in the health system. This situation might be partially explained by decades of poor influence from the central level followed by years of top-down management. Despite this, the experience demonstrates that it is possible to improve health district regulation by conditioning the financial support to a more rational use of available resources. It also shows that the population uses public health services if they are functional and if their costs are fair, affordable, and predictable.

Our fourth and final objective was to discuss the appropriateness of shifting from fee-for-service to flat rate payments as a policy option for countries facing similar financial problems in their attempts to develop more equity in their financing system. The Kisantu experience has shown that acting upon one of the elements of the system can, if designed using a systems thinking approach, contribute to rebalance the entire system. It also showed that the implementation of flat fees cannot be an isolated measure or a goal in itself, but must be part of a more comprehensive package of activities in the field of rationalization of human resources and of diagnostic and therapeutic behavior.

## Conclusions

Rural populations from DRC are facing increasing barriers to access health care of good quality. This situation is fundamentally related to the context of commercialization of health care caused by the association of a fee-for-service payment system with a generalized lack of regulation throughout the country. Between 2008 and 2011, the BTC health program launched a set of reforms in the Kisantu district in the province of Bas Congo, with the aim of improving access to health care for its population. The rationalization of the use of resources and the regulation of the financing of the health services were at the core of those reforms. A conditionally subsidized flat fee system at the GH was implemented, and the existing flat fee system at the IHC was reorganized. These important measures were accompanied by other reforms, such as human resources development and rationalization of activities, the implementation of rational prescription tools, and support of health care prepayment schemes.

We used an action-research process that included a systems thinking approach, in which the complexity of the health system and the unexpected or unintended consequences of the reforms were managed through an iterative and dynamic process and adaptive solutions.

The results were immediate in terms of access to quality health care. The hospitalization rate, first-level curative care utilization rate, referral rate from the IHCs to the GH, and proportion of recovery of patient bills all improved. The relevance of referral (proportion of true positives) also improved, while the average length of stay and competition between the hospital and the first level decreased.

The action-research process proved to be particularly adapted to the implementation of changes within an open complex adaptive system such as the health care system. In addition, it gradually generated a capacity-building process for the district management team regarding leadership and its understanding of the complexity of the health system. The Kisantu experience also demonstrates that a systems thinking approach is essential to address complex issues, such as the lack of access to quality health care.

## References

[1] World Health Organization (2008). World health report – primary health care now more than ever.

[2] World Health Organization (2010). World health report – health systems financing. The path to universal coverage.

[3] Gilson L, Beattie A, Doherty J, Gilson L, Lambo E, Shaw P, The World Bank (1996). Sustainable health care financing in Southern Africa. The lesson of user fee experience in Africa.

[4] Kegels G (1994). Paying for health care instead of buying drugs. An experience from western Mali. Ann Soc Belge Med Trop.

[5] Walt G, Pavignani E, Gilson L, Buse K (1999). Health sector development: from aid coordination to resource management. Health Policy Plan.

[6] Soeters R, Peerenboom P, Mushagalusa P, Kimanuka C (2011). Performance-based financing experiment improved health care in the Democratic Republic of Congo. Health Aff.

[7] Gilson L, Hanson K, Sheikh K, Akua I, Ssengooba F, Bennett S (2001). Building the field of health policy and systems research: social science matters. PLoS Med.

[8] Hercot D, Meessen B, Ridde V, Gilson L (2011). Removing user fees for health services in low-income countries: a multi-country review framework for assessing the process of policy change. Health Policy Plan.

[9] Meessen B, Gilson L, Tibouti A (2011). User fee removal in low-income countries: sharing knowledge to support managed implementation. Health Policy Plan.

[10] Nabyonga J, Mugisha F, Kirunga C, Macq J, Criel B (2011). Abolition of user fees: the Uganda Paradox. Health Policy Plan.

[11] Nimpagariste M, Bertone M (2011). The sudden removal of user fees: the perspective of a frontline manager in Burundi. Health Policy Plan.

[12] Ridde V, Haddad S (2009). Abolishing user fees in Africa. PLoS Med.

[13] Ridde V, Morestin F (2011). A scoping review of the literature on the abolition of user fees in health care services in Africa. Health Policy Plan.

[14] Ministry of Public Health, DRC (2010). Plan National de Développement Sanitaire.

[15] Grodos D, Tonglet R (2004). Le district sanitaire urbain en Afrique subsaharienne: enjeux, pratiques et politiques.

[16] Coghlan B, Brennan RJ, Ngoy P, Dofara D, Otto B, Clements M (2006). Mortality in the Democratic Republic of Congo: a nationwide survey. Lancet.

[17] Fund for Peace (2009). Failed states index: most vulnerable countries.

[18] United Nations Development Program (2009). International human development indicators.

[19] Ministry of Public Health, DRC (2008). Rapport sur les Comptes Nationaux de la Santé.

[20] Ministry of Public Health, DRC (2008). Costing et planification des ressources de services de santé dans le cadre de la mise en æuvre de la stratégie de renforcement du système de santé.

[21] World Bank: Democratic Republic of Congo Health sector rehabilitation support project.

[22] Ministry of Public Health, DRC (2006). Stratégie de Renforcement du Système de Santé.

[23] Ministry of Public Health, DRC (2010). Stratégie de Renforcement du Système de Santé.

[24] Ministry of Public Health, DRC (2011). Plan National de Développement des Ressources Humaines pour la Santé.

[25] Wembonyama S, Mpaka S, Tshilolo L (2007). Médecine et santé en République Démographique du Congo: de l'indépendance à la 3ème république. Med Trop.

[26] Lamboray JL, Laing C (1984). Partners for better health. World Health Forum.

[27] Criel B, Van Baelen H (1993). Paying for the Kasongo Hospital in Zaire: a conceptual framework. Health Policy Plan.

[28] Walt G, Shiffman J, Schneider H, Murray SF, Brugha R, Gilson L (2008). Doing’ health policy analysis: methodological and conceptual reflections and challenges. Health Policy Plan.

[29] Van Olmen J, Criel B, Van Damme W, Marchal B, Van Belle S, Van Dormael M, Van Lerberghe W, Kegels G, De Brouwere V (2010). Studies in health services organisations and policy. Analysing health systems to make them stronger.

[30] Eisenhardt K (1989). Building theories from case study research. Acad Manage Rev.

[31] Nitayarumphong S, Mercenier P (1994). Ayutthaya research project: Thailand experiences on health systems research.

[32] Grodos D, Mercenier P, Van Lerberghe W, Kegels G, De Brouwere V (2000). La recherche sur les systèmes de santé: Mieux comprendre la méthodologie pour mieux agir. Studies in health services organisations and policy.

[33] Holmström J, Ketokivi M (2009). Bridging practice and theory. A design science approach. Decis Sci.

[34] Atun R (2012). Health systems, system thinking and innovation. Health Policy Plan.

